# Role of diffuse low-level heteroplasmy of mitochondrial DNA in Alzheimer’s disease neurodegeneration

**DOI:** 10.3389/fnagi.2015.00142

**Published:** 2015-07-23

**Authors:** Tiziana Casoli, Liana Spazzafumo, Giuseppina Di Stefano, Fiorenzo Conti

**Affiliations:** ^1^Center for Neurobiology of Aging, INRCA IRCCSAncona, Italy; ^2^Center of Biostatistics, INRCA IRCCSAncona, Italy; ^3^Department of Experimental and Clinical Medicine, Section of Neuroscience and Cell Biology, Università Politecnica delle MarcheAncona, Italy

**Keywords:** Alzheimer’s disease, mtDNA, mutation, aging, allele, MitoChip, heteroplasmy

## Abstract

Alzheimer’s disease (AD) is the most common form of dementia in the elderly. The vast majority of cases are not linked to a known genetic defect and the molecular mechanisms underlying AD pathogenesis are still elusive. Evidence suggests that mitochondrial dysfunction is a prominent feature of the disease, and that mitochondrial DNA (mtDNA) alterations may represent a possible starting point of the pathophysiological cascade. Although specific mtDNA alterations have been reported in AD patients both in brain and peripheral tissues, such as D-loop mutations, 4977-bp deletion and poly-C tract D310 cytosine insertion, a generalized subtle allelic shift has also been demonstrated. This shift is significant for a few nucleotide positions (nps), but it is also detectable for most nps, although at a lower level. As single allelic substitutions can unlikely be determinant, it is proposed that the combination of all of them could lead to a less efficient oxidative phosphorylation, thus influencing AD development and course.

## Introduction

Alzheimer’s disease (AD) is an age-related neurological disorder that begins with occasional memory loss, indistinguishable from memory disturbances associated with physiological aging, and soon develops into a severe and debilitating disease, characterized by confusion, aggressiveness, anxiety, sleep disturbance, depression, aberrant motor behavior and severe cognitive impairments (Fernández et al., 2010). AD behavioral symptoms are a direct consequence of the decimation of neurons, and researchers worldwide are trying to unveil the cause of the widespread neuronal death. Many have focused on “plaques” and “tangles”. Plaques are toxic aggregates of amyloid-ß (Aß) peptide distributed in the neuropil; tangles are intraneuronal accumulations of the hyper-phosphorylated tau protein in twisted filaments. Although much progress has been made regarding the knowledge of inter- and intra-molecular interactions of both plaques and tangles (Perl, 2010), the reason why they develop or why only some individuals are susceptible is still elusive. A popular, though controversial, view posits that Aß is upstream of tau in AD pathogenesis and triggers the conversion of tau from a normal to a toxic state (Bloom, 2014), implying that Aß is central in the pathology (Herrup, 2015; Musiek and Holtzman, 2015). The observation that AD is associated with a number of systemic manifestations, including altered gene expression (Miller et al., 2008), oxidation (Moreira et al., 2005), and changes in enzymatic activity (Inestrosa et al., 1994), indicates that there could be common factors underlying both central and peripheral manifestations (Morris et al., 2014).

Mitochondrial dysfunction precedes both central nervous system (CNS) neuropathological changes and peripheral dysfunction, and it is one of the best documented systemic alteration in AD (Galindo et al., 2010; Leuner et al., 2012; Selfridge et al., 2013). Mitochondria involvement has been confirmed by *in vitro* studies using cybrid models, i.e., mitochondria-depleted cell lines replenished with mitochondria from patients with AD (Sheehan et al., 1997). Cybrids mimic many features of AD, including decreased cytochrome c oxidase (COX) activity, increased oxidative stress and Aß levels, and cell death activation (Khan et al., 2000). The relationship between impaired mitochondrial function and Aß neuropathology is not well understood, although some evidence suggests that mitochondrial bioenergetics and brain metabolism affect amyloid precursor protein (APP) processing (Brody et al., 2008; Xiang et al., 2010). Indeed, inhibition of energy metabolism promotes potentially amyloidogenic pathways and amyloidosis (Gabuzda et al., 1994; Gasparini et al., 1997). Most mitochondrial functions are reportedly impaired in AD, and the instability and limited repairability of mitochondrial DNA (mtDNA), due to absence of histones and reduced efficacy of enzymatic repair system, as well as the crucial role it plays in oxidative phosphorylation (OXPHOS), render mtDNA a candidate to be the primary site of damage. mtDNA is inherited exclusively by maternal lineage and both mtDNA haplotypes and maternal family history are associated with cognitive and cerebrospinal fluid (CSF) biomarkers in AD patients (Honea et al., 2012; Ridge et al., 2013). Interestingly, mtDNA accumulates mutations throughout the aging process (Michikawa et al., 1999; Krishnan et al., 2007), which is indeed the main risk factor for AD (Swerdlow et al., 2014). Here we shall explore different aspects of mitochondrial alterations found in AD patients, with special emphasis on mtDNA, reporting new data on subtle widespread changes in nucleotide sequence.

## Variations of Mitochondrial Function in Alzheimer’s Disease

Mitochondria exhibit robust changes in both brain and peripheral cells of AD patients. Fluorodeoxyglucose (FDG) Positron Emission Tomography (PET) studies show that the severity of dementia correlates closely to the magnitude of brain metabolism reduction, and that decreased glucose metabolism in cortical areas of AD subjects precedes functional decline. On these bases, FDG PET investigations are being increasingly adopted to assist clinicians in AD diagnosis, and to predict future cognitive deterioration. Metabolic decline can be attributed to reduced expression of mitochondrial and nuclear genes encoding subunits of the tricarboxylic acid (TCA) cycle and mitochondrial electron transport chain enzymes. Indeed, α-ketoglutarate dehydrogenase, pyruvate dehydrogenase, and COX expression and/or activity are reduced in AD (Gibson et al., 1998; Maurer et al., 2000). COX activity is also decreased in platelets (Parker et al., 1994), and represents one of the most well-documented systemic change reported in AD. Interestingly, a recent study shows reduced platelet COX activity in cognitively healthy subjects with a maternal AD history compared to those with a paternal history of AD. This implies that COX abnormalities reflect the maternal inheritance and possibly mtDNA involvement, which derives exclusively from the mother (Mosconi et al., 2011).

Dysfunctional mitochondria contribute also to calcium dyshomeostasis through impaired buffering capacity (Supnet and Bezprozvanny, 2010). Both mitochondria and endoplasmic reticulum (ER) are involved in altered Ca^2+^ homeostasis described in AD, resulting in increased cytosolic Ca^2+^ concentration (Peterson et al., 1985). Mitochondria-associated endoplasmic reticulum membrane (MAM) is a specialized subdomain of the ER membrane that regulates ER-mitochondria communications, and its function is significantly increased in fibroblasts from patients with AD (Area-Gomez et al., 2012). Strictly connected to mitochondrial calcium buffering derangement is the activation of apoptotic pathway. Indeed, a transient increase in mitochondrial Ca^2+^ induces opening of permeability transition pore (PTP), and apoptosis. Although it remains controversial whether apoptosis plays a major role in AD neurodegeneration, many components of the apoptotic pathway are activated or altered in AD brains and peripheral cells. Studies in lymphocytes of sporadic AD patients report significant changes in apoptotic markers compared to age-matched controls, including increased DNA fragmentation, enhanced vulnerability to proapoptotic stimuli, and increased levels of caspases 3, 8 and 9 (Tacconi et al., 2004; Leuner et al., 2012). Lymphocytes from AD patients bearing one or two APOE ε4 alleles exhibit a higher rate of apoptotic cell death and caspase 3 activation than non-ε4 carriers (Frey et al., 2006).

In addition, mitochondrial dynamics is altered in AD. The brain of patients with AD shows morphological mitochondrial alterations, such as reduced number, increased size and broken internal membrane cristae (Selfridge et al., 2013). Size and number of mitochondria are regulated by the dynamic processes of fusion and fission and recent studies reported significant changes in the expression of almost all mitochondrial fusion and fission related proteins in brains from AD patients, including dynamin-like protein 1 (DLP1), optic atrophy 1 (OPA1), and fission 1 (Fis1; Wang et al., 2009, 2012). The major effect of these modifications is reduced fission, which leads to structurally damaged and swollen mitochondria in vulnerable areas of the CNS.

Overall, mitochondria undergo important alterations in both brain and peripheral tissues in AD, supporting the hypothesis of a systemic dysfunction involving energy metabolism.

## Mitochondrial DNA Modifications in Alzheimer’s Disease

A central role in mitochondrial dysfunctions is played by mtDNA, as it represents a semi-permanent and transmissible site of injury. mtDNA is composed of a circular molecule and the sequence codes for the proteins of OXPHOS complexes, and the tRNAs and rRNAs used for the synthesis of mtDNA-encoded proteins. It is present in multiple copies within a mitochondrion, and therefore it is possible that mutant and wild-type molecules coexist in a single cell. During the aging process mtDNA mutations accumulate in cells and tissues, especially in those with active oxidative metabolism like the brain (Lin et al., 2002; Kennedy et al., 2013). To date, two mechanisms have been proposed to explain this observation: oxidative damage deriving from exposition to reactive oxygen species (ROS) and mtDNA replication errors. Numerous studies show an age-related rise of oxidative damage to lipids, proteins and DNA (Smith et al., 1991; Ward et al., 2005; Santos et al., 2013), and a progressive increase in 8-hydroxyguanine (8-OHdG) levels was reported in mtDNA with aging (Mecocci et al., 1997). However, antioxidant enzymes neither prevent the increase of mutations during aging, nor their use increases lifespan (Lagouge and Larsson, 2013), suggesting that an alternative mechanism may determine mtDNA damage, e.g., accumulation of duplication errors. This process has been investigated in the mtDNA mutator mice that have a defect in the proofreading function of the mtDNA polymerase PolG leading to a progressive and random collection of mtDNA point mutations (Trifunovic et al., 2004). These mice show a 3- to 5-fold increase of the level of point mutations, as well as premature aging and reduced lifespan. Questioning ROS as central causative agents in age-dependent accumulation of mtDNA mutations reflects the notion that, despite their bad reputation, ROS are important signaling molecules involved in metabolism regulation, cell differentiation and stress response (Hou et al., 2014), and that their effects could even be beneficial in stimulating internal anti-oxidant defense response. The increase in mtDNA mutations observed during aging is exacerbated in AD patients and in AD mouse models, in which a further increase of mtDNA mutations is present in both brain and peripheral tissues (Coskun et al., 2012; Kukreja et al., 2014). Coskun et al. (2004) demonstrated that mtDNA control region displays more sporadic mutations in AD brains than in control cases, and that some of them are specific for AD patients like T414C and T477C. The levels of mtDNA 4977-bp deletion are increased in AD brains during the early stages of the disease compared to controls and decline later, probably due to the death of the affected cells (Corral-Debrinski et al., 1994). Other peculiarities of mtDNA in AD have been reported, such as the presence of the 7028C allele and a higher frequency of the poly-C tract D310 with a number of cytosine >7 (Coto et al., 2011). Conversely, conclusive evidence for an association of mtDNA common haplogroups with AD has not been provided to date (Hudson et al., 2012).

## Low-Level Heteroplasmic Sequence Changes of Mitochondrial DNA

**Figure 1:**
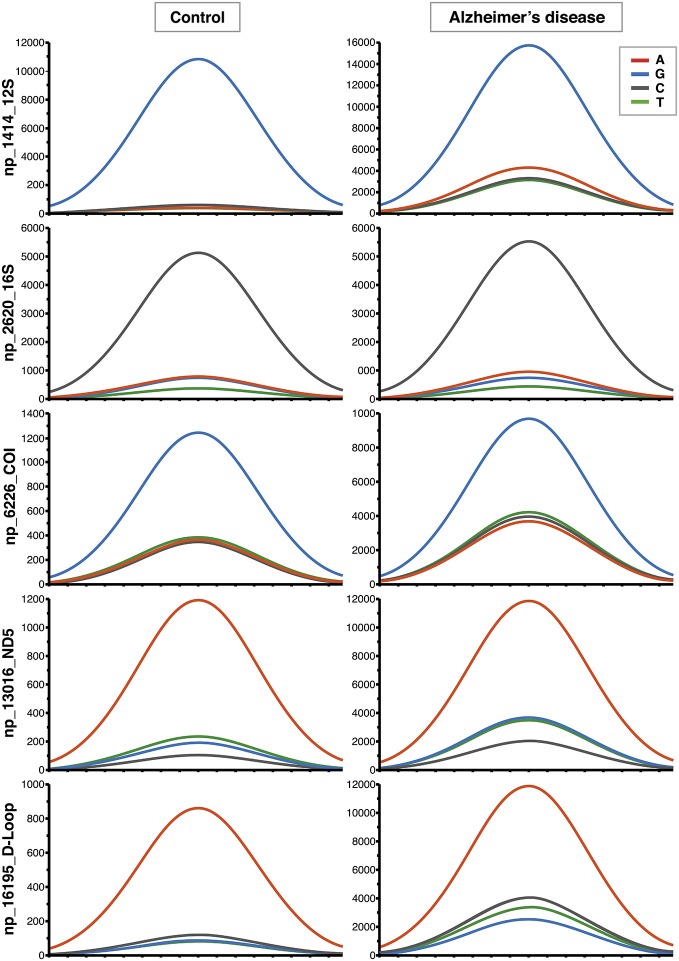
**Increased contribution of non-reference alleles in mitochondrial DNA (mtDNA) of Alzheimer’s disease (AD) subjects.** Representative Affymetrix sequencing chromatograms (forward strand) relating to 5 of the 270 nucleotide positions (nps) significantly different between controls and AD patients. The highest peak corresponds to the reference allele. The increased contribution of the other three non-reference alleles can be seen in AD group.

**Figure 2:**
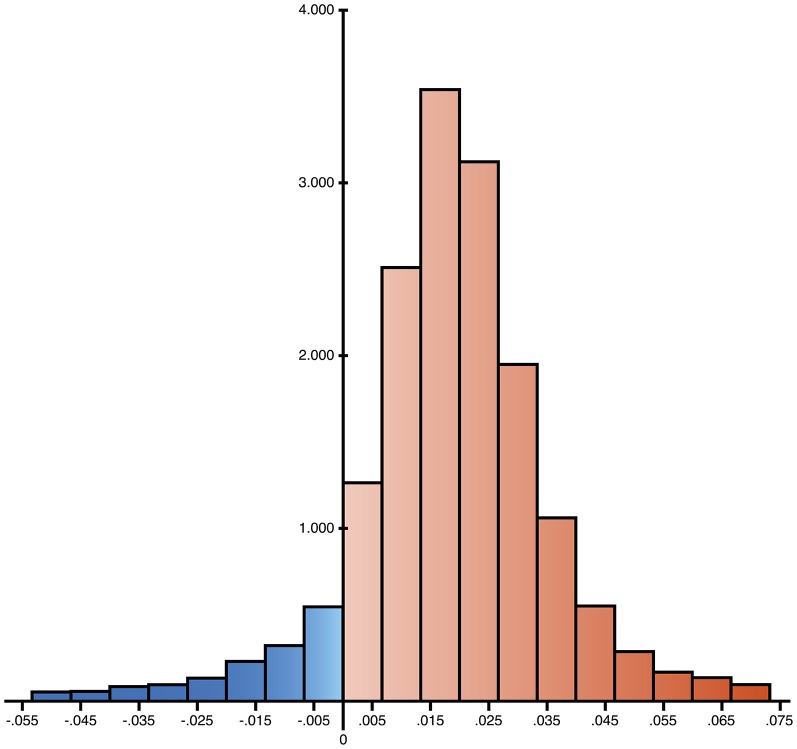
**Distribution of the differences of ratio of expected allele (REA) values between controls and AD group.** 16,544 nps have been analyzed and the differences resulted positive for 14,847 cases, reported in red in the histogram (89.7%), suggesting a widespread increase of allelic shift in AD patients.

mtDNA mutations could involve few copies or all the molecules present in an organism, and these two conditions are called heteroplasmy or homoplasmy, respectively. An homoplasmic mutation could determine the specific mtDNA haplogroup, whereas heteroplasmy influences the phenotype depending on the associated damage and on the percentage of copies affected. The percentage threshold for a critical phenotype vary for different types of mutation. The threshold for deleted mtDNA is approximately 50–60%, while for point mutations it is 70–90% (Schon et al., 2012). A particular category of heteroplasmic mutations includes those with a low percentage of mutated copies, i.e., low-level heteroplasmic mutations. Their detection is problematic because the signal coming from the minor component cannot be easily distinguished from background noise even with the latest Next Generation Sequencing (NGS) technology. We recently employed Affymetrix MitoChip v2.0 array to sequence mtDNA from whole blood of AD patients and controls (Casoli et al., 2014). MitoChip is a mtDNA resequencing array with eight 25-mer probes/base position (four oligonucleotide probes/strand) corresponding to the whole revised Cambridge Reference Sequence (rCRS). Each 25-mer probe is varied at the central position to incorporate each possible nucleotide (A, G, C, or T), and fluorescence data are elaborated by an algorithm. We set “model type” at diploid to enable the detection of heteroplasmy and “quality score threshold” at 3 to provide the best base calling accuracy and rate. Resequencing software GSEQ 4.1 returns two kinds of output files: single nucleotide polymorphism (SNP) View and Probe Intensity allowing the identification of single base alterations, but not insertions or deletions. SNP View files display the base calls only for bases with mutations, classified as homoplasmic or heteroplasmic (resembling the diploid condition with about 50% heteroplasmy), compared to the reference bases of rCRS. Probe intensity files provide values of fluorescence intensity corresponding to all of the four bases for each nucleotide position (np) for both sense and antisense filament, producing a quantitative estimate of allelic contribution. The analysis of SNP View files revealed that neither homoplasmic nor heteroplasmic total mutations were significantly different in the control as compared to AD group. On the other hand, analysis of the Probe Intensity files identified 270 significantly different nucleotide positions (nps), which, with one exception, showed an increased contribution of non-reference alleles in AD patients, as defined by ratio of expected allele (REA) values, calculated as the log ratio of the signal intensity of the reference allele at any site, as indicated in the rCRS, to the average signal intensity of the other three alleles (Coon et al., 2006). This result demonstrates that controls have a notably higher fidelity to reference base than AD patients, which consequently show a significant increase of allelic shift. The 270 nps were characterized by low-level heteroplasmy in AD subjects (5–20%; Figure 1), were not associated with homo- or heteroplasmic mutations, did not belong to a particular gene or class of genes, and could not be ascribed to a particular nucleotide suggesting that that the observed allelic shift was random and unspecific. The coding nps (167) included 81% of non-synonymous mutations, therefore, although single substitution unlikely could be determinant, the combination of all of them could really influence disease development and course. Interestingly, the increased allelic shift observed in AD was not only restricted to the 270 significant nps, but concerned the majority of the mtDNA nps analyzed. Indeed, mean REA values were higher in controls in 89.7% of the 16,544 nps analyzed (Figure 2). A control sequence tiled on the array, corresponding to a plasmid DNA (TagIQ-EX), showed mean REA values higher for controls in 44.1% of cases, indicating that the sequencing results were not affected by experimental biases. The final outcome of these observations is that mtDNA involvement in AD should not be searched in single base mutation or individual gene malfunctioning, but rather in random and widespread damage that probably leads to less efficient OXPHOS and altered cell metabolism. To date, it is not possible to state whether these changes are inherited or acquired, but considering the age-dependent accumulation of mtDNA mutations (Larsson, 2010) and the fact that aging is the most important risk factor for AD, it is conceivable that the allelic alterations accumulate during the lifetime at a rate depending on baseline mitochondrial function and environmental factor exposure (Swerdlow et al., 2014). This notion by no means rules out the possibility that inherited mutations may contribute to brain aging (Ross et al., 2013), as suggested by the recent demonstration that low heteroplasmy variants can be maternally inherited, and that they accumulate mostly in postmitotic tissue such as mucle (Avital et al., 2012; Guo et al., 2013).

## Conclusion and Future Directions

Here, we discussed new findings on the involvement of mtDNA in AD, and report that diffuse, low-level heteroplasmic sequence changes are important features of AD pathology. mtDNA damage can be considered an upstream event in this disease, and may induce alterations of OXPHOS enzymes. Many researchers have ascribed this damage to progressive oxidation of mtDNA molecules by ROS that are potentially mutagenic and highly concentrated in its proximity. However, the failure of clinical trials aimed at modulating ROS in AD by antioxidants (Freund-Levi et al., 2006; Lloret et al., 2009) has stimulated a partial revision of this assumption. Physical activity and dietary restrictions are effective in preventing age-associated diseases like AD, but both of them actually determine a transient increase of ROS production instead of a decrease, and it is possible that increased release of oxidative species leads to enhanced stress defense. It therefore seems that the best strategy against ROS is an exposure in an on/off mode, which may stimulate endogenous reactions. Since mtDNA mutations have been reported in most age-related neurodegenerative diseases (Cha et al., 2015), a step forward should be taken to verify if the reported mtDNA diffuse allelic shift is specific for AD, by comparing groups of patients affected by different types of neurodegeneration. The proved involvement of mitochondria in AD neurodegeneration may also stimulate the search for interventions aimed at targeting these organelles. Various molecules could be targeted to mitochondria thereby providing a basis for the design of new medications, including RNA or DNA (e.g., antisense oligonucleotides, ribozymes, plasmid DNA expressing mitochondrial genes etc.).

## Conflict of Interest Statement

The authors declare that the research was conducted in the absence of any commercial or financial relationships that could be construed as a potential conflict of interest.
